# The effect of inositol hexaphosphate on the expression of selected metalloproteinases and their tissue inhibitors in IL-1β-stimulated colon cancer cells

**DOI:** 10.1007/s00384-012-1445-3

**Published:** 2012-03-15

**Authors:** Małgorzata Kapral, Joanna Wawszczyk, Magdalena Jurzak, Andrzej Hollek, Ludmiła Węglarz

**Affiliations:** Department of Biochemistry, Medical University of Silesia, 41-200 Sosnowiec, Narcyzow 1, Poland

**Keywords:** IP6, Inflammation, Metastases, Colon cancer, Real-time QRT-PCR

## Abstract

**Introduction:**

Matrix metalloproteinases (MMPs) have repeatedly been shown to play a very active role in extracellular matrix degradation associated with tumor invasion and metastasis. Tissue inhibitors of MMPs (TIMPs) are well-known for their ability to inhibit MMP activity thereby inhibiting malignant progression. Inositol hexaphosphate (IP6 phytic acid) has been recognized to have both preventive and therapeutic effects against various cancers including that of colon. In in vitro studies, IP6 has been demonstrated to inhibit cancer cell adhesion and migration. In the present study, the effect of IP6 on the expression of MMP and TIMP genes was evaluated in unstimulated and IL-1β-stimulated colon cancer cell line Caco-2.

**Materials and methods:**

Real-time QRT-PCR was used to validate the transcription level of selected MMP and TIMP genes in Caco-2 cells after treatment with 1 ng/ml of IL-1β, 2.5 mM of IP6, and both for 6, 12, and 24 h.

**Results:**

Stimulation of cells with IL-1β only resulted in an overexpression of MMP and their TIMP mRNAs. A significant decrease in MMP-13, MMP-3, MMP-2, and TIMP-1 basal expression was achieved by IP6. IP6 was also an efficient downregulator of MMP-1, MMP-9, and TIMP-2 genes transcription stimulated by IL-1β in 6 h lasting culture. After 12 h, IL-1β-induced MMP-2 mRNA expression was significantly reduced by IP6.

**Conclusion:**

Proinflammatory cytokine IL-1β upregulates MMP and TIMP mRNAs expression in colon cancer epithelial cells Caco-2. IP6 (2.5 mM) influences constitutive expression of both MMP and TIMP genes and downregulates IL-1β stimulated transcription of some of these genes. IP6 exerts its anti-metastatic activity through modulation of MMP and TIMP genes expression to prevent cancer cell migration and invasion.

## Introduction

Chronic inflammation is associated with a high cancer risk. Inflammatory mediators are also present in the microenvironment of tumors epidemiologically unrelated to inflammation. Smouldering inflammation in local environment of tumors induces many tumor-promoting effects. Cancer-related inflammation contributes to proliferation and survival of malignant cells, angiogenesis and metastasis, and represents a target for therapeutic strategies [1]. Pro-inflammatory cytokines, such as interleukin-1 (IL-1), IL-6, IL-8, and tumor necrosis factor-α (TNF-α), actively participate in the promotion and progression phases of carcinogenesis [2]. IL-1 is produced by components of tumor microenvironment, such as tumor and stromal cells, and infiltrated inflammatory/immune cells [3]. IL-1 is known to be upregulated in many tumor types and it has been implicated as an essential factor in tumor progression by influencing the expression of metastatic and angiogenic genes. In various experimental models and in cancer patients, the increased local levels of IL-1 usually correlate with tumor invasiveness [4].

Tumor growth, invasion, and metastasis are multistep processes that include cell proliferation, proteolytic digestion of the extracellular matrix (ECM), cell migration through basement membranes to reach the circulatory system, and establishment of new proliferating colonies at the metastatic sites [5]. Cancer cells’ invasion through the basement membranes must be preceded by their detachment from both neighboring cells and the surrounding matrix. Thus, a critical event in tumor cell invasion is degradation of a complex network of extracellular macromolecules such as collagen, proteoglycans, fibronectin, and laminin that act as a barrier to the spread of cancer cells to distal sites [6, 7].

The majority of matrix degradation is carried out by the matrix metalloproteinases (MMPs), a family of zinc-dependent neutral endopeptidases that are collectively capable of degrading the different components of ECM. The human MMP gene family consists of several subgroups of structurally related members, such as collagenases (MMP-1, MMP-8, MMP-13, MMP-18), gelatinases (MMP-2, MMP-9), stromelysins (MMP-3, MMP-10, MMP-11), matrilysins (MMP-7, MMP-26), enamelysins (MMP-18, MMP-19), metalloelastases (MMP-12), membrane-type metalloproteinases (MMP-14, MMP-15, MMP-16, MMP-17, MMP-24, MMP-25), and others. These enzymes have been implicated in the process of tumor growth, invasion, and metastasis [6, 8]. Numerous studies in a variety of tumor types, including colon carcinoma, demonstrated overexpression of MMPs in malignant tissues in comparison to adjacent normal tissues. The increased MMP expression and activity have been observed to correlate with advanced tumor stage, increased invasion and metastasis, and shortened survival [9–11]. Furthermore, an accumulating evidence suggests that MMPs may play an important role in the tissue degradation observed in inflammatory bowel disease [12, 13].

MMP activity is counterbalanced by endogenous inhibitors, such as specific tissue inhibitors of matrix metalloproteinases (TIMPs). A family of TIMPs consists of structurally related proteins (TIMP-1, TIMP-2, TIMP-3, and TIMP-4), revealing different tissue and cell type specific expression and regulation patterns [14, 15]. They exert a dual control on the MMPs by inhibiting both the active form of the MMPs and their activation process. TIMPs inhibit MMPs by 1:1 stoichiometric binding and an imbalance between the activation and inhibition of MMP activity in favor of the MMP activity is believed to play an important role in the pathophysiology of cancer by facilitating the invasion of tumor cells through the ECM [14].

Overexpression of TIMP-1 has been observed to inhibit some tumor growth and suppress metastatic ability of cancer cells. Elevated TIMP levels are reported in association with cancer progression and identified as poor prognostic indicators in several human tumor types such as colorectal cancer, breast cancer, prostate, and lung cancer [16, 17].

Cancer prevention strongly acknowledges the importance of diet as dietary factors are the most important environmental risk factors for cancer [18]. Within recent several years, a large number of naturally occurring health-enhancing substances of plant origin have been recognized to have beneficial effects on cancers including colon cancer and other disorders of the gastrointestinal tract. These biologically active plant chemicals are now known as phytochemicals or nutraceuticals [19, 20].

A hexaphosphorylated inositol (IP6) carbohydrate is a major fiber-associated component of wheat bran, cereals, legumes, grains, seeds, and nuts and is also widely distributed in animal cells and tissues at concentrations of about 5–100 μM [21, 22]. Epidemiological studies have shown that IP6-rich dietary factors have been associated with reduced cancer risk and incidence of mortality due to colorectal cancer [23, 24]. It was noticed that only fiber with high IP6 content showed negative correlation with colon cancer, indicating that it could be IP6 and not fiber that suppressed colon cancer [22]. Moreover, pure IP6 has reproducibly been demonstrated to have both preventive and therapeutic effects against various cancers in experimental models including that of colon [25, 26]. Anticancer actions of IP6 have been postulated to involve boosting immunity, antioxidant properties, anti-apoptotic effects, and signal transduction reducing cell proliferation and inducing differentiation of malignant cells [27, 28]. IP6 has been demonstrated to prevent and abrogate both primary tumor and metastasis in vivo [29]. It also inhibited metastasis in vitro by affecting MDA-MB 231 human breast cancer cell adhesion to fibronectin and collagen. The loss of cell adhesion induced by IP6 and attributed to growth arrest and cell death conferred a less malignant phenotype. IP6 significantly inhibited the secretion of MMP-9 from MDA-MB 231 cells as well as significantly reduced the invasion properties of cancer cells in vitro [30]. In colorectal cancer cell line Caco-2 IP6 modulated MMP-2, TIMP-1, and TIMP-2 genes expression. In long lasting culture along with increasing IP6 concentrations, the cells expressed lower and lower MMP-2 mRNA level. In response to IP6, an increased transcriptional activity of the TIMP-2 gene was accompanied by a decrease in TIMP-1 gene transcription [31].

The aim of the present study was to examine the influence of IP6 on the transcriptional activity of selected MMPs of their three subgroups, i.e., MMP-1, MMP-2, MMP-3, MMP-9, MMP-10, MMP-13, and that of their TIMP-1 and TIMP-2 in human colon cancer Caco-2 cells as well as IP6 ability to affect IL-1β-influenced changes in mRNA levels of the studied genes.

## Material and methods

### Cell culture

The Caco-2 human colon adenocarcinoma cells (DSMZ, Braunschweig, Germany) were routinely cultured in RPMI 1640 medium (Sigma Aldrich) supplemented with 10% fetal bovine serum (GibcoBRL), 100 U/ml penicillin and 100 μg/ml streptomycin (both from Sigma Aldrich), and 10 mM HEPES (GibcoBRL). They were maintained at 37°C in a 5% CO_2_ atmosphere. For experiments, the cells were seeded at the density of 1 × 10^6^ per 5 ml medium in 21.5 cm^2^ culture flasks. After 3 days, the media containing serum were changed to serum-free media and the cells were cultured for 24 h. They were then stimulated with 1 ng/ml IL-1β (Sigma Aldrich) for 30 min. Afterwards, the cells were treated with 2.5 mM IP6 as dipotassium salt (Sigma Aldrich; P5681; distilled water dissolved and pH 7.4 adjusted) for 6, 12, and 24 h. In separate cultures, cells were incubated with IL-1β or IP6 at the indicated concentrations and for the indicated times. The untreated Caco-2 cells were used as the control.

### RNA extraction

Total RNA was extracted from control and treated with IL-1β and IP6 cells with the use of TRIZOL® reagent (Invitrogen) following the protocol of the manufacturer. Integrity of the RNA extracts was qualitatively checked by electrophoresis in 1.0% agarose gel stained with ethidium bromide. RNA concentration was determined spectrophotometrically on the basis of absorbance values at a wavelength of 260 nm using a GeneQuant pro (Amersham Biosciences).

### Real-time QRT-PCR assay

Detection of the expression of MMP and TIMP mRNAs was carried out using a QRT-PCR technique with a SYBR Green chemistry (SYBR Green Quantitect RT-PCR Kit, Qiagen) and Opticon™ DNA Engine Continuous Fluorescence detector (MJ Research) as described previously [31]. Oligonucleotide primers specific for MMP-1, MMP-2, MMP-3, MMP-9, MMP-10, MMP-13, TIMP-1, and TIMP-2 mRNAs were designed using Primer Express 2.0 software (PE Applied Biosystems, USA; Table 1). QRT-PCR assay was performed in triplicate for each sample. The thermal profile for one-step RT-PCR was as follows: 50°C for 30 min for reverse transcription and 95°C for 15 min followed by 45 cycles at 94°C for 15 s, 55°C for 30 s, and 72°C for 45 s for amplification. Following RT-PCR, the samples were subjected to temperature ramp from 60°C to 95°C at the rate of 0.2°C/s with continuous fluorescence monitoring for melting curve analysis. The mRNA copy numbers of examined genes were determined on the basis of the commercially available standard of β-actin (TaqMan DNA Template Reagent Kit, Applied Biosystems). The obtained results of mRNA copy number were recalculated per μg of total RNA. The specificity of RT-PCR reaction was confirmed by determining the characteristic temperature of melting for each amplimer and by 6% polyacrylamide gel electrophoresis of RT-PCR products with their visualization using silver staining.

**Table 1:** Characteristics of primers used in experiment

Gene	Primer sequence	Product amplified (bp)	TM (°C)
MMP1	F: 5′-GCTCATGAACTCGGCCATTCTCTTGGACT-3′	281	81
	R: 5′-CGGGTAGAAGGGATTTGTGCGCATGTA-3′		
MMP2	F: 5′-TCCACTGTTGGTGGGAACTCA-3′	121	83
	R: 5′-TGGTCGCACACCACATCTTT-3′		
MMP3	F: 5′-CCGAGAAATGCAGAAGTTCCTTGGATTGG-3′	131	82
	R: 5′-TCGGGATGCCAGGAAAGGTTCTGAAGTG-3′		
MMP9	F: 5′-TTCTGCCCCAGCGAGAGA-3′	101	81
	R: 5′-GTGCAGGCGGAGTAGGATTG-3′		
MMP10	F: 5′ TCTTGCATTCCTTGTGCTGTTG 3′	101	81
	R: 5′ ATTGCTGGGCAAGATCCTTGT 3′		
MMP13	F: 5′-ACACCTACACCGGCAAAAGC-3′	121	79
	R: 5′-CATTTGTCTGGCGTTTTTGGA-3′		
TIMP1	F: 5′-GCCATGGAGAGTGTCTGCGGATACTTCC-3′	118	86
	R: 5′-GCCACGAAACTGCAGGTAGTGATGT-3′		
TIMP2	F: 5′-CCCCAAGCAGGAGTTTCTCGACATCG-3′	100	82
	R: 5′-TGGACCAGTCGAAACCCTTGGAGGCT-3′		

*bp* base pair, *TM* temperature of melting

### Statistical analysis

Statistical analysis was carried out using the Statistica PL 9.0 software. The one-way ANOVA followed by post hoc Tukey’s test were applied to evaluate differences in the expression of examined genes between untreated cells and cells treated with IP6 and IL-1β. All the results are expressed as means ± SD. Values of *p* < 0.05 were considered as statistically significant.

## Results

### The basal mRNA expression of matrix metalloproteinases of collagenase, gelatinase, and stromelysin subgroups and of their tissue inhibitors in Caco-2 human colon adenocarcinoma cells

**Fig. 1:**
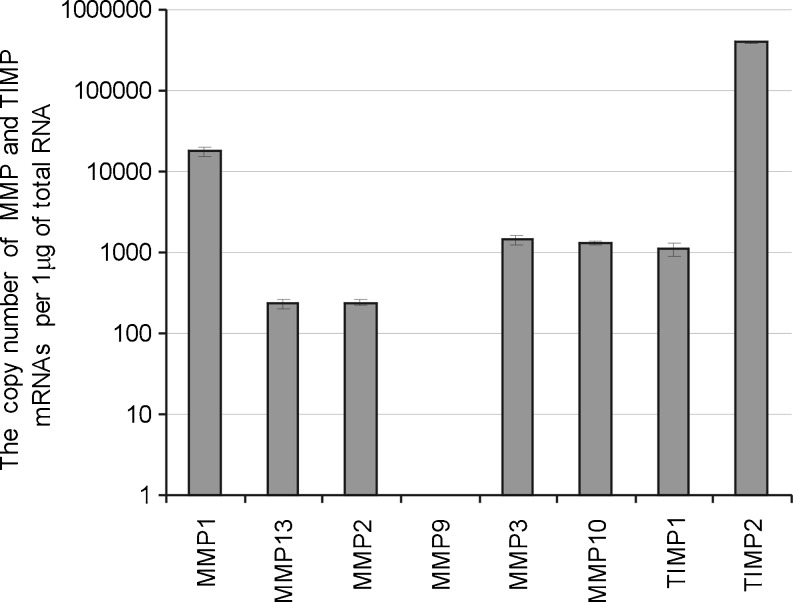
The basal expression of MMP and TIMP mRNAs in Caco-2 cells.The cells were seeded at the density of 1 × 10^6^ and grown as described in the “Materials and methods” section. The real-time QRT-PCR was performed for MMP and TIMP genes. The Caco-2 did not show the expression of MMP-9 gene. *Graph* represents the mean ± SD of three individual experiments

The effect of IL-1β and IP6 on the expression of mRNA of MMP-1, MMP-2, MMP-3, MMP-9, MMP-10, and MMP-13 and that of their tissue inhibitors TIMP-1 and TIMP-2 in colon cancer cells using real-time QRT-PCR assay was determined. The transcriptional activity of all examined MMP genes except that of MMP-9 was detected in unstimulated Caco-2 cells. The cells also exhibited the expression of both TIMPs under standard conditions. The highest level of transcription was manifested by MMP-1 and TIMP-2 genes (Fig. 1).

### The influence of IL-1β on MMP and TIMP genes expression

**Fig. 2:**
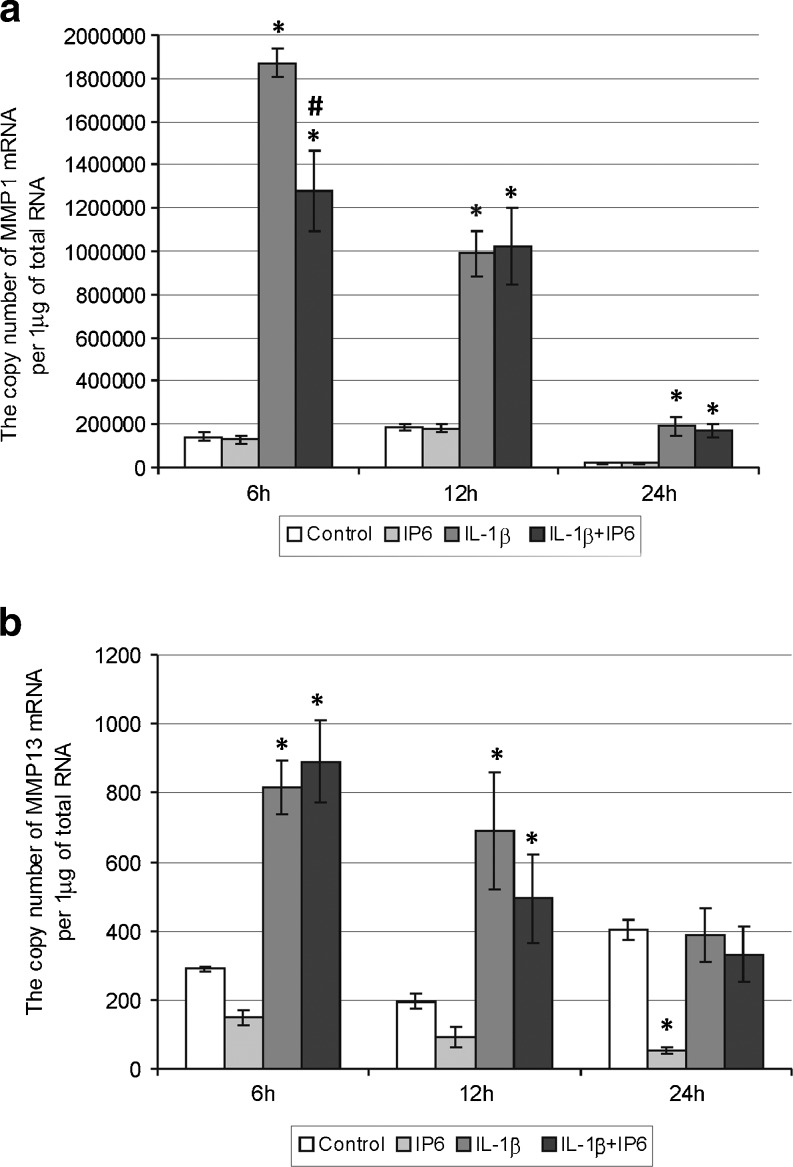
Expression of genes encoding collagenases in Caco-2 cells as determined by real-time RT-PCR. Comparison of the copy number of **a** MMP-1 and **b** MMP-13 mRNAs per 1 μg of total RNA in Caco-2 cells treated with 2.5 mM IP6 and 1 ng/ml IL-1β for 6, 12, and 24 h (the results are presented as mean ± SD of three separate experiments; *p < 0.05 vs control Caco-2 cells; *#p* < 0.05 vs IL-1β-stimulated cells)

**Fig. 3:**
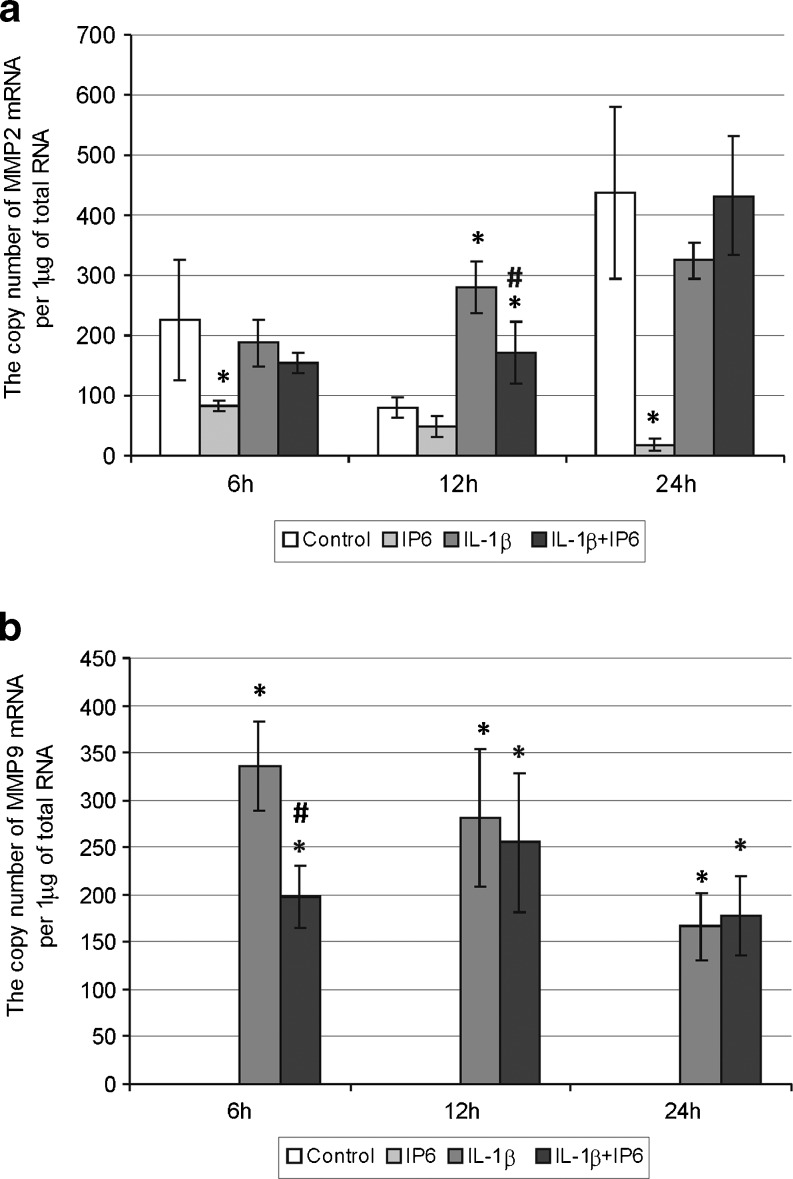
Expression of genes encoding gelatinases in Caco-2 cells as determined by real-time RT-PCR. Comparison of the copy number of **a** MMP-2 and **b** MMP-9 mRNAs per 1 μg of total RNA in Caco-2 cells treated with 2.5 mM IP6 and 1 ng/ml IL-1β for 6, 12, and 24 h. In Caco-2, cells did not detect the expression of MMP-9 gene either under normal conditions or following treatment with 2.5 mM IP6 for each interaction time (the results are presented as mean ± SD of three separate experiments; *p < 0.05 vs control Caco-2 cells; *#p* < 0.05 vs IL-1β-stimulated cells)

**Fig. 4:**
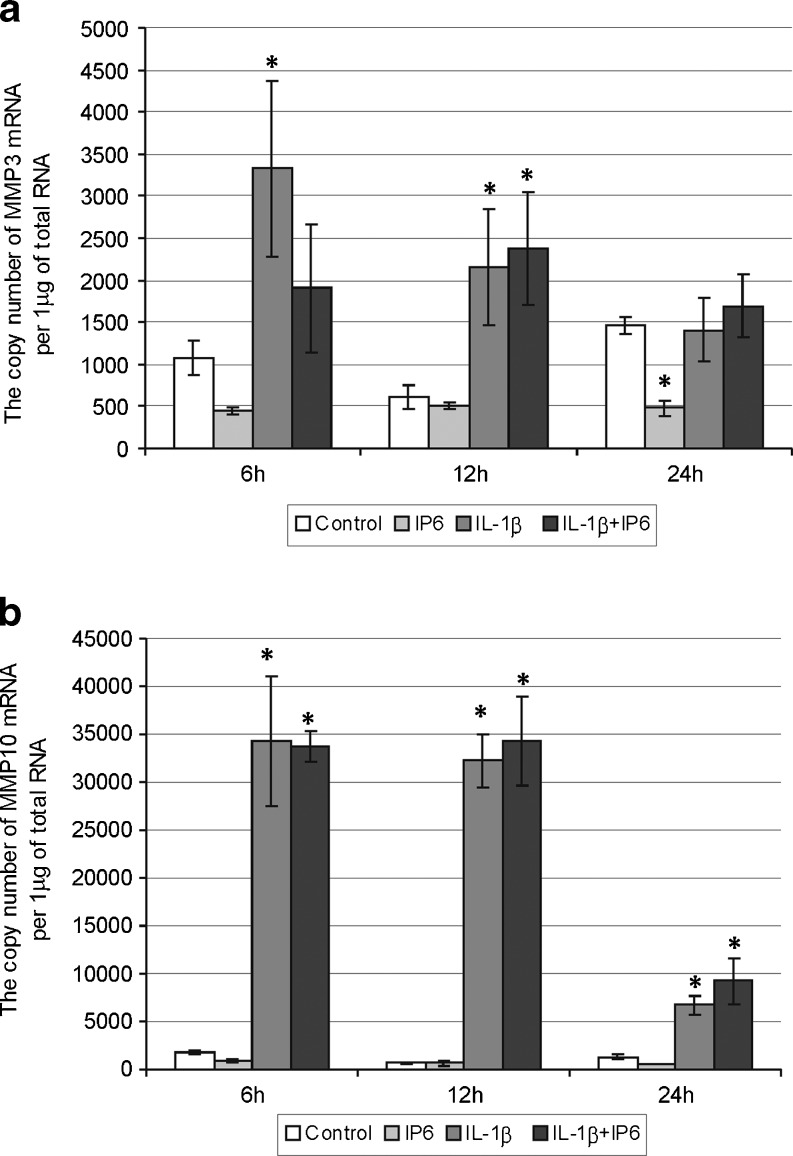
Expression of genes encoding stromelysins in Caco-2 cells as determined by real-time RT-PCR. Comparison of the copy number of **a** MMP-3 and **b** MMP-10 mRNAs per 1 μg of total RNA in Caco-2 cells treated with 2.5 mM IP6 and 1 ng/ml IL-1β for 6, 12, and 24 h (the results are presented as mean ± SD of three separate experiments; *p < 0.05 vs control Caco-2 cells; *#p* < 0.05 vs IL-1β-stimulated cells)

**Fig. 5:**
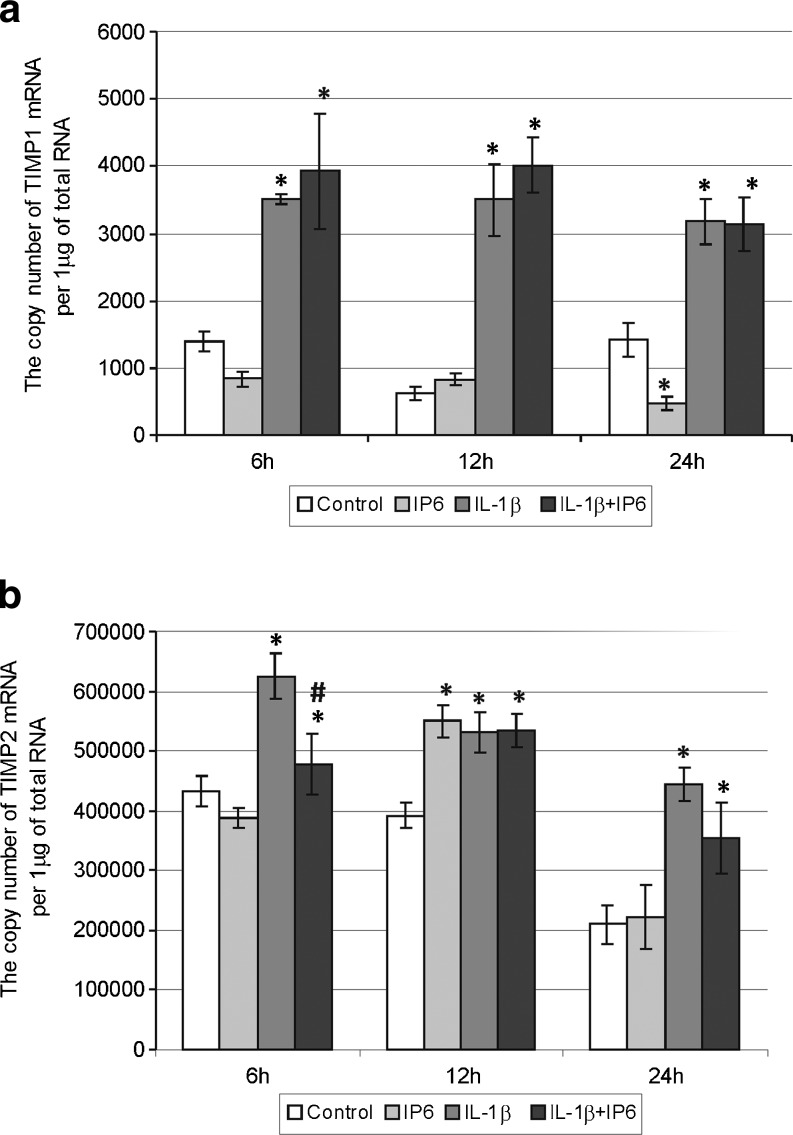
Expression of genes encoding tissue inhibitors of matrix metalloproteinases in Caco-2 cells as determined by real-time RT-PCR. Comparison of the copy number of **a** TIMP-1 and **b** TIMP-2 mRNAs per 1 μg of total RNA in Caco-2 cells treated with 2.5 mM IP6 and 1 ng/ml IL-1β for 6, 12, and 24 h (the results are presented as mean ± SD of three separate experiments; *p < 0.05 vs control Caco-2 cells; *#p* < 0.05 vs IL-1β-stimulated cells)

Stimulation of Caco-2 with 1 ng/ml of IL-1β for 6–24 h resulted in an upregulation of MMP-1, MMP-9, MMP-10, TIMP-1, and TIMP-2 genes as compared with untreated cells (*p* < 0.05) (Figs. 2a, 3b, 4b, and 5). A significantly higher MMP-13 and MMP-3 mRNA levels were determined following IL-1β cell treatment for 6–12 h than in controls (Figs. 2b and 4a). The MMP-2 expression was markedly induced in response to IL-1β after 12 h (Fig. 3a).

### Changes in constitutive and IL-1β-stimulated expression of genes encoding collagenases in Caco-2 cells treated with IP6

In a time course experiment, 2.5 mM IP6 had no influence on MMP-1 expression (*p* > 0.05; Tukey test; Fig. 2a). Statistical analysis revealed significant decrease in the MMP-1 gene expression in cells exposed to IL-1β and IP6 for 6 h compared to the cultures treated with IL-1β only (*p* < 0.001; Tukey test). IP6 downregulated constitutive transcriptional activity of MMP-13 gene in Caco-2 and a statistically significant difference in this gene expression between control and IP6-treated cells was observed after 24 h (*p* < 0.001; Tukey test). No marked changes in MMP-13 gene activity were demonstrated in IL-1β-stimulated cells and those treated with IL-1β and IP6 for 6–24 h (Fig. 2b).

### Changes in constitutive and IL-1β-stimulated expression of genes encoding gelatinases in Caco-2 cells treated with IP6

A comparison of expression of genes encoding gelatinases in the control cells and cultures treated with IP6 and IL-1β for 6–12–24 h showed significant differences (Fig. 3). Cells incubated with 2.5 mM IP6 for 6 (*p* = 0.049), 12 (*p* = 0.715), and 24 h (*p* = 0.002) presented decreased MMP-2 transcript level in comparison to control cells. At time points of 6 and 24 h, transcriptional activity of this gene in cultures treated with IL-1β only and those incubated with both IL-1β and IP6 revealed similar levels (*p* > 0.05). After 12 h, statistically significant decrease in mRNA values for MMP-2 was observed in cells exposed to IL-1β and IP6 in relation to cells treated with IL-1β only (*p* = 0.0212; Fig. 3a).

Caco-2 cells did not reveal the expression of MMP-9 gene under both basal conditions and following treatment with IP6 at the analyzed time intervals. However, IL-1β strongly induced MMP-9 gene expression in the cells and in short-lasting cultures (6 h), IL-1β-induced transcription of this gene was remarkably downregulated by IP6 (*p* = 0.0019). There were no differences in MMP-9 transcript level between IL-1β-stimulated cells and cells exposed to IL-1β and IP6 for both 12 and 24 h (Fig. 3b).

### Changes in constitutive and IL-1β-stimulated expression of genes encoding stromelysins in Caco-2 cells treated with IP6

Treatment of Caco-2 cells with 2.5 mM IP6 for 24 h resulted in statistically significant decrease in MMP-3 gene expression in comparison to control cells (*p* = 0.0096). The levels of MMP-3 mRNA in IL-1β-stimulated cells and cells treated with IL-1β and IP6 revealed no statistically significant differences after 6–12–24 h (Fig. 4a), indicating no influence of IP6 on enhanced expression of MMP-3 promoted by IL-1β. Furthermore, MMP-10 gene transcription appeared to be highly responsive to stimulatory action of IL-1β and IP6 had no effect on the basal transcriptional activity of this gene (*p* > 0.05). Statistically insignificant differences in mRNA levels of MMP-10 in cells exposed to IL-1β alone and treated with IL-1β/IP6 for 6–24 h were noted (Fig. 4b).

### Changes in constitutive and IL-1β-stimulated expression of genes encoding tissue inhibitors of matrix metalloproteinases in Caco-2 cells treated with IP6

The level of TIMP-1 mRNA expression was significantly lower in Caco-2 cells incubated with IP6 for 24 h than in untreated cells (*p* = 0.0158). However, 12 h incubation of cells with IP6 resulted in higher level of TIMP-2 mRNA compared to control (*p* = 0.0007). The TIMP-2 gene expression increased by IL-1β at 6 h was significantly downregulated by IP6 (*p* = 0.0039; Fig. 5).

## Discussion

The family of matrix metalloproteinases and their tissue inhibitors play an essential role in a variety of pathologic and physiologic conditions. MMPs can degrade essentially all extracellular matrix components and thus, contribute to every stage of tumor progression including tumor cell invasion and metastasis. [8, 32]. The activity of MMPs is regulated at several levels, but many researches underline that transcriptional regulation of the genes expression beside the activation of latent proenzyme forms and interaction with TIMPs could be considered most important mechanism [12, 32, 33]. Thus, it is thought that the local balance of MMP expression and activation versus the level of TIMP determines the extent of tissue destruction mediated by MMPs. Studies have shown that MMP protein concentrations correlate well with mRNA expression in vitro [12]. Therefore, in the present study, we concentrated our attention on MMP and TIMP genes expression at transcriptional level. Quantitative mRNA expression of MMP-1, MMP-2, MMP-3, MMP-9, MMP-10, MMP-13, and TIMP-1 and TIMP-2 was evaluated simultaneously by real-time RT-PCR in human colon cancer cells Caco-2.

MMP transcripts are frequently expressed at low or undetectable levels but their level substantially increases in tissues undergoing remodeling, such as in inflammation and cancer [33]. The main source of MMPs in most tumors is stromal fibroblasts. Moreover, inflammatory cells infiltrating tumor as well as inflamed colonic mucosa also produce MMPs. Recruitment of these cells appears to be largely enhanced by MMPs produced within the tumor environment [34]. Moreover, inflammatory cells secrete cytokines which enhance MMPs expression by tumor and stromal cells [35]. IL-1β, besides IL-6 and TNF-α, is the most abundantly expressed cytokine, both in normal and inflamed mucosa [36]. Activation of protein kinase C (PKC), mitogen-activated protein kinases (MAPKs), activator protein-1 (AP-1), and nuclear factor κB (NF-κB) is suggested to be an essential event in the cascade leading to the stimulation of MMPs and TIMPs gene expression in response to proinflammatory cytokines (IL-1β, TNF-α) [35, 37, 38].

The results of the present study revealed a distinct pattern of basal and IL-1β-stimulated expression of MMPs and TIMPs mRNA in human colon carcinoma cells. Under normal conditions, Caco-2 cells constitutively expressed genes encoding MMP-1, MMP-2, MMP-3, MMP-10, MMP-13, and their tissue inhibitors TIMP-1 and TIMP-2, whereas MMP-9 transcription was not detected. This observation can be referred to the work of Gan et al. [39], which demonstrated detectable but very low levels of MMP-9 mRNA in Caco-2 cells in the absence of inflammatory stimuli. Among examined MMPs and their inhibitors, we detected the highest amounts of collagenase-1 (MMP-1) and TIMP-2 mRNAs. MMP-1 is expressed by a variety of tumor cells, where its levels are constitutively high even in the absence of apparent external stimuli [40, 41]. The collagenases are the foremost enzymes capable of cleaving triple helix of collagens type I, II, III, and V into fragments subsequently degraded by other MMPs, such as gelatinases [35, 41]. Bendardaf et al. [41] suggested that the intense expression of interstitial collagenases, such as MMP-1, is required, mostly in the initial stages of tumor invasion and metastasis because tumor invasion is facilitated by degradation of interstitial stroma, the main component of ECM.

The addition of IL-β (1 ng/ml) to the cell cultures resulted in enhancement of all MMPs as well as TIMPs transcript levels in a time-dependent manner. IL-1β was capable of activating the transcription of TIMPs and each of MMPs except MMP-2 after 6 h, and overexpression of MMP-2 gene was noted after 12 h stimulation. The differences in the activation time of MMP genes may be explained by the fact that those of them inducible by extracellular stimuli (MMP-1, MMP-3, MMP-7, MMP-9, MMP-10, MMP-12, and MMP-13) possess an AP-1 transcription factor binding site in the promoter. On the contrary, MMP-2, MMP-11, and MMP-14 genes do not contain AP-1 elements [35]. The human MMP-2 gene promoter contains AP-2 transcription factor binding site [35, 42].

Our observation on MMP expression remains in agreement with other findings. Kermorgant et al. [43] confirmed the presence of MMP-2 and MMP-1 but not MMP-9 mRNA in Caco-2 cells by RT-PCR. In the absence of inflammatory stimuli, Caco-2 cells expressed low levels of MMP-9 mRNA. Stimulation of cells with IL-1β induced the production of MMP-9 [39]. Claramunt et al. [44] found that in untreated Caco-2 cells, MMP-2 was the predominant gelatinase, whereas MMP-9 was negligible one. However, cytokine treatment resulted in a significant dose-dependent upregulation of MMP-9 activity in those cells. On the contrary, MMP-2 remained unchanged by the cytokine treatment. The authors concluded that MMP-9 is inducible and its gene promoter region is highly responsive to most growth factors and cytokines, whereas MMP-2 is expressed constitutively by most cells and appears to be moderately induced or repressed. Salmela et al. [45] demonstrated that IL-1β at 24 h upregulated MMP-7 and MMP-10 expression in HT-29, WiDr, and Caco-2 gut epithelial cell lines. In Caco-2 cells, IL-1β increased collagenase-1 expression at least threefold compared to unstimulated control samples. Roeb et al. [9] postulated that inhibiting overexpression of MMPs may be a useful preventive or therapeutic adjunct in colorectal cancer. According to the opinion of Brinckerhoff et al. [40], signal transduction pathways participating in MMP gene expression have lead to new therapeutic strategies of cancer prevention and treatment. In the last years, many drugs aiming at MMP downregulation have been synthesized and are undergoing clinical trials in various human cancers [46, 47]. Inhibitors of MMPs (MMPIs) fall into three pharmacologic species: collagen peptidomimetics, tetracycline derivatives, and bisphosphonates [14]. Peptidomimetic MMP inhibitors are pseudopeptide derivatives that mimic the structure of collagen at the site where MMP binds to cleave it. The inhibitor binds reversibly at the active site of the MMP in a stereospecific manner and chelates the zinc with, in most cases, a hydroxamic acid functional group, on the enzyme activation site. The earliest generation of these inhibitors (batimastat) had low water solubility and was not orally available. The next generation of inhibitors (marimastat, prinomastat) was orally available, but they were frequently linked with musculoskeletal toxicity, probably due to their off-target effects on non-MMP metalloproteinases. These agents are relatively nonspecific, inhibiting the activity of MMP-1, MMP-2, MMP-3, MMP-7, and MMP-9 [14, 47]. Another group of MMPIs are tetracyclines (tetracycline, doxycycline, and minocycline) and its chemically modified derivatives (metastat), which do not possess antibiotic activities. These compounds effectively inhibit a broad range of MMPs through their ability to chelate Zn^2+^ ion on the active site of enzymes, thus changing their interactions with substrates; it can also downregulate the transcription of MMPs mRNA. [48, 49]. These agents inhibit both gelatinases and collagenases. The bisphosphonates are a synthetic compounds with a high affinity for the hydroxyapatite crystal of bone. These drugs are used in instability of calcium homeostasis and for the palliation and prevention of bone metastases in patients with breast cancer and multiple myeloma. The agent exerts the effect on MMPs via inhibition of their enzymatic activity [14]. Pharmacological inhibition of MMPs relies on the ability of drugs to chelate the metal ions. Their property makes them nonselective inhibitors targeting multiple MMPs, since the Zn^2+^ is present in the catalytic domain of all MMPs, so broad range blockade of multiple MMPs would not be expected to be useful [50]. The lack of therapeutic success of MMP inhibitors in cancer treatment has largely been due to toxicity of the drugs on normal tissues as well as conflicting roles in both promoting and reducing tumor metastastasis [51]. Moreover, the drugs were not effective because they were employed too late, in advanced, metastatic stage of cancer [47, 52]. Currently, there are used highly specific monoclonal antibodies against MMPs; however, the difficulty of producing macromolecular proteins and the parenteral administration of drugs limit their therapeutic potential [53].

**Fig. 6:**
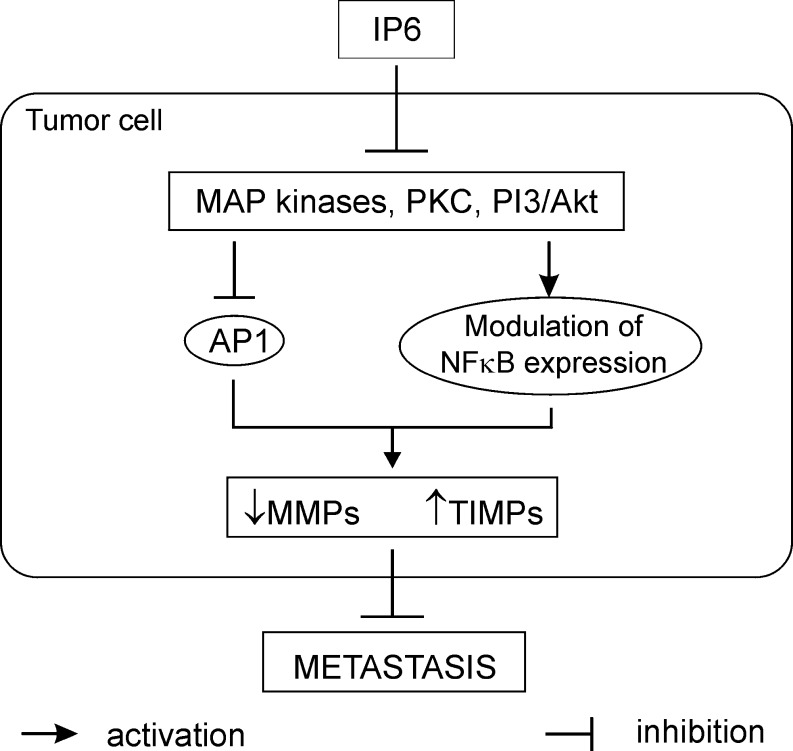
The potential mechanism of IP6 action on preventing or reducing metastasis of cancer cells

The great potential for chemoprevention and therapeutic achievement could have agents capable of suppressing numerous intracellular signaling pathways [54]. Inositol hexaphosphate, a natural dietary component, exerts anti-cancer influence on tumor cells via phosphatidylinositol-3 kinase (PI3K), MAPK, PKC, AP-1, and NFκB (Fig. 6). It has been shown to block PKC and PI3K and inhibit AP-1 as well as MAPK activation [22, 26, 55]. Our previously published data demonstrated that IP6 modulated the expression of p65 subunit of nuclear factor κB and its IκBα inhibitor in the intestinal epithelial cells [56]. Thus, IP6 may exhibit its own action on key elements of MMP genes expression pathways both in untreated and stimulated by interleukin-1β.

The main aim of the current study was to evaluate the effect of phytic acid on the expression of genes encoding metalloproteinases and their tissue inhibitors in IL-1β-treated colon cancer Caco-2 cells. Studies concerning the influence of IP6 on the expression of MMPs and TIMPs in IL-1β-stimulated cells have not yet been published. A recent experiment by Claramunt and co-workers [44] has demonstrated that curcumin, naturally occurring nutriceutical, was able to significantly downregulate MMP-9 activity in Caco-2 cells stimulated by pro-inflammatory cytokines. As observed in the present study, treatment of Caco-2 cells with 2.5 mM IP6 for 24 h showed markedly decreased MMP-13 and MMP-3 genes expression in comparison to control. IP6 also influenced a decrease in MMP2 transcript level after 6 and 24 h incubation. The level of TIMP-1 gene expression in cells exposed to IP6 for 24 h was lower than in untreated cells, and at 12 h incubation with IP6, the cells exhibited higher level of TIMP-2 mRNA. IP6 counteracted the stimulatory effect of IL-1β on MMP-1, MMP-9, and TIMP-2 genes expression in Caco-2 cells by decreasing their mRNA levels in 6 h lasting cultures. After 12 h, IL-1β-induced MMP-2 mRNA expression was remarkably downregulated by IP6.

In conclusion, the current data suggest that proinflammatory cytokines of which IL-1β is the major representative may upregulate MMP and TIMP expression in gut epithelial cells which indicates that inflammatory reactions leading to cytokine release may stimulate MMPs and their inhibitors expression these cells. MMPs could then contribute to intestinal tissue destruction and propagation of inflammation and invasive capacity of tumor cells. Moreover, inositol hexaphosphate at the concentration of 2.5 mM was able to inhibit IL-1β-induced MMP-1, MMP-2, MMP-9, and TIMP-2 genes expression at transcriptional level in Caco-2 cells. In addition, the decrease in MMP-2, MMP-3, MMP-13, and TIMP-1 basal transcripts level could be evoked by 2.5 mM IP6 after relatively longer time of its action. Thus, it can be suggested that IP6 may exert its anti-metastatic activity through modulation of MMP and TIMP genes expression to prevent cancer cell migration and invasion.

Further investigation on IP6 is clearly warranted, the more that as a naturally occurring product, in contrast with conventional anti-cancer agents, IP6 offers safety and demonstrates specific activity on tumor cells without affecting the normal cells. Thus, inositol hexaphosphate could be used not only in promotion but also in all stages of cancer progression by downregulation of matrix metalloproteinases expression.
